# Association between hip pain and radiographic hip osteoarthritis in primary care: the CHECK cohort

**DOI:** 10.3399/BJGP.2021.0547

**Published:** 2022-09-21

**Authors:** Guido AM Rondas, Erin M Macri, Edwin HG Oei, Sita MA Bierma-Zeinstra, Hanneke BM Rijkels-Otters, Jos Runhaar

**Affiliations:** Erasmus MC University Medical Center Rotterdam, Rotterdam.; Department of Orthopaedics and Sports Medicine, Erasmus MC University Medical Center Rotterdam, Rotterdam.; Department of Radiology and Nuclear Medicine, Erasmus MC University Medical Center Rotterdam, Rotterdam.; Department of General Practice and Department of Orthopedics & Sports Medicine, Erasmus MC University Medical Center Rotterdam, Rotterdam.; Department of General Practice, Erasmus MC University Medical Center Rotterdam, Rotterdam.; Department of General Practice, Erasmus MC University Medical Center Rotterdam, Rotterdam.

**Keywords:** epidemiology, hip joint, hip osteoarthritis, pain, radiographic osteoarthritis

## Abstract

**Background:**

The diagnosis of hip osteoarthritis (OA) is often based on clinical symptoms, such as pain and stiffness, and radiographic features. However, the association between hip pain and hip radiographic OA (ROA) remains uncertain.

**Aim:**

To examine the association between hip pain and hip ROA.

**Design and setting:**

Cross-sectional analysis of a Dutch cohort, the Cohort Hip and Cohort Knee (CHECK) study.

**Method:**

The participants (aged 45–65 years) had all experienced hip and/or knee pain for which they had not had a prior consultation or were within 6 months of their first consultation with a GP. Using weight-bearing anteroposterior pelvis radiographs, definite and early-stage hip ROA were defined as Kellgren and Lawrence grade ≥2 and ≥1, respectively. Presence of ROA and pain was assessed in the hips of all participants. The association between hip pain and ROA was assessed using generalised estimating equations.

**Results:**

The prevalence of definite ROA was 11.0% (*n* = 218/1982 hips), with prevalence in painful and pain-free hips of 13.3% (*n* = 105/789) and 9.5% (*n* = 113/1193), respectively. Prevalence of early-stage hip ROA was 35.3% (*n* = 700/1982), with prevalence in painful and pain-free hips of 41.2% (*n* = 325/789) and 31.4% (*n* = 375/1193), respectively. Compared with pain-free hips, the odds ratio painful hips was 1.51 (95% confidence interval [CI] = 1.16 to 1.98) for definite ROA and 1.47 (95% CI = 1.24 to 1.75) for early-stage ROA.

**Conclusion:**

Hip pain was associated with definite and early-stage hip ROA, yet the overall ROA prevalence was modest and the prevalence among pain-free hips was substantial. Therefore, radiographs provided little assistance with help to identify patients with hip OA among patients who recently presented with hip or knee complaints.

## INTRODUCTION

Hip osteoarthritis (OA) is a common cause of morbidity among older people that is associated with hip pain and stiffness, and impaired mobility.^1^ Although less prevalent than knee OA, the global all-age symptomatic hip OA prevalence in 2010 was 0.85%^2^ and the European prevalence in adults aged ≥60 years was 7%–8%.^3^^,^^4^ OA currently accounts for up to 2.5% spent of the gross national product in Western countries, mainly attributable to knee and hip arthroplasties costs.^4^^,^^5^ In patients aged ≥45 years, 4% of primary care consultations are registered with an OA International Classification of Diseases code^6^ and the overall primary care hip pain consultation rate is approximately 13/1000 consultations.^7^ On account of an ageing world population, hip OA prevalence and costs are expected to increase.^8^

Pain is the most reported symptom among patients with hip OA.^1^^,^^4^ It is often pain and the associated functional disability, participation restriction, and loss of independence that pushes patients to seek health care for a GP.^2^^,^^9^^–^^11^ Consequently, hip pain is a key clinical feature used to classify or diagnose hip OA in primary care.^12^^,^^13^ In primary care, the use of radiographic imaging in the diagnostic process is strongly discouraged, as there is a general mismatch between radiographic signs of OA and patients’ symptoms; radiographic findings are thought not to influence the choice of treatment strategy by the GP, and are not predictive for the course of symptoms.^4^^,^^13^^,^^14^ However, these recommendations to not use radiography for OA diagnosis are purely based on data obtained about knee OA, and OA in the hip joint is thought to be distinctively different from OA in the knee joint.^15^

The association between hip pain and hip radiographic OA (ROA) remains uncertain as relevant literature is scarce and inconsistent.^16^^–^^19^ In prior research, definitions for definite and severe ROA have differed considerably, as have pain definitions. Hence, the odds ratios (ORs) have differed substantially, ranging from 1.6 to 123.4 for severe ROA and 1.3 to 2.8 for definite ROA.^16^^,^^17^^,^^19^ As the symptomatic hip ROA prevalence was low in these studies, results were imprecise. Additionally, in one study only participants aged >60 years were analysed, and another analysed these associations only in males because of an absence of severe ROA in females.^18^^,^^19^ Also, pain was related to definite ROA in both sexes in one study,^18^ whereas in another study it was associated with severe ROA in males, but not in females.^17^

**Table:** How this fits in

The diagnosis of hip osteoarthritis (OA) is often based on a combination of clinical symptoms, such as pain and stiffness, and radiographic features. Previous research investigating the association between hip pain and hip radiographic OA (ROA) is limited and results conflicting. In this cross-sectional study, hip pain was only modestly associated with hip ROA in patients with early symptomatic hip OA. GPs should consider implementing the first steps of OA treatment in patients with clinically suspected hip OA. This study affirms that referral of these patients for radiographic confirmation of the diagnosis of hip OA is not necessary.

Many scientific studies on hip OA therapies, including conservative interventions (for example, exercise therapy^20^), analgesics,^21^ and experimental studies on potential disease-modifying drugs,^22^ restrict the recruitment of patients to those with confirmed structural changes to the hip joint, visible on radiographs. In the absence of knowledge on the true association between hip symptoms and hip ROA, these studies might target the wrong structures (as many individuals with radiographic changes might not have pain) and potentially exclude many patients with hip symptoms (because of the absence of radiographic changes) that do require medical attention.

The aim of this study was to examine the cross-sectional association between hip pain and prevalent hip ROA in a Dutch cohort study of middle-aged males and females with hip and/or knee pain.

## METHOD

### The CHECK study

The Cohort Hip and Cohort Knee (CHECK) study is a prospective cohort study of 1002 participants with a 10-year follow-up (inclusion 2002–2005). The study protocol and sample have been described previously.^23^^,^^24^ In brief, individuals were eligible if they:

- had knee and/or hip pain or stiffness;
- were aged 45–65 years; and
- were enrolled within 6 months of their first visit to the GP for this complaint or had not yet sought care for these complaints.

Exclusion criteria were:

- any other pathological condition that could cause hip or knee complaints (for example, other rheumatic disease, previous hip or knee joint replacement, congenital dysplasia, osteochondritis dissecans, intra-articular fractures, septic arthritis, Perthes’ disease, ligament or meniscus damage, plica syndrome, or Baker’s cyst);
- any comorbidity precluding physical assessment or follow-up;
- malignancy in the past 5 years; or
- the inability to understand the Dutch language.

As well as through GPs, participants were also recruited through daily newspapers or the Dutch Arthritis Society website. All CHECK participants gave written informed consent and the study was approved by the medical ethics committees of all participating hospitals.

For the present study, data from the first visit (‘baseline’) were used, and included all participants with a complete set of radiographs.

### Hip pain

Participants were enrolled if they reported pain and/or stiffness in one or both hips and/or knees. During the baseline examination, participants verbally confirmed (yes/no) in which joints they experienced pain and/or stiffness, and body weight and height were measured to calculate body mass index (BMI). Using questionnaires, information on age (years), duration of complaints (months), ethnicity (‘White’ versus ‘other’), education level (‘primary’, ‘secondary’, or ‘higher’), presence of morning stiffness (yes/no), any pain medication use (yes/no), pain severity (0–10), and Western Ontario and McMaster Universities Osteoarthritis Index (WOMAC) pain, function, and stiffness scores (all 0–100) was collected.

### Radiographic assessment

At baseline, weight-bearing anteroposterior pelvis radiographs of all participants were acquired. Both hips were graded according to the Kellgren and Lawrence (K&L) scale.^25^ A grade was assigned, ranging from 0–4, based on the presence and severity of osteophytes, joint space narrowing, sclerosis, and bone-end deformity, in which 0 suggests no ROA and grade 4 suggests severe ROA. Grading was performed by trained readers. A detailed description of the scoring approach has been published.^26^

For the main outcome definite hip ROA was defined as a K&L ≥2. Additionally, a secondary outcome was evaluated, early-stage radiographic hip OA, defined as K&L ≥1. Hips with missing baseline K&L scores were excluded from analyses.

### Statistical analyses

The analyses were performed using IBM SPSS Statistics (version 25). Means, corresponding standard deviations (SDs), and frequencies for baseline characteristics were calculated, including hip pain and ROA prevalence. The association between self-reported hip pain (yes/no) and both definite and early-stage hip ROA was evaluated, using generalised estimating equations, to account for the two hips included for each participant. No covariates were included as the aim was to evaluate whether self-reported hip pain was predictive for hip ROA, not whether ROA was causally related to hip pain. A sample size ≥432 would provide ≥80% power for a relative risk ≥2 for an assumed hip pain prevalence of 10% in hips free of ROA.^27^

Post hoc sensitivity analyses were then performed. First, as the difference between sexes was unclear in prior research, a sex-stratified analysis was performed using the *Z*-test statistic^28^^,^^29^ to test for differences in associations between males and females (secondary analysis I). Second, the analyses were repeated investigating self-reported hip pain and ROA in a more strictly defined control group, containing only participants without any self-reported hip pain, and thus excluding participants with unilateral hip complaints. This was done because having contralateral hip pain might increase the risk of having hip ROA in these unilateral hip complaints (secondary analysis II). Finally, as knee pain is associated with hip ROA,^17^ analyses were repeated only in participants who also reported pain in at least one knee (sensitivity analysis III), and only in participants who reported no knee pain (sensitivity analysis IV).

## RESULTS

Baseline radiographic K&L grades of 11 participants (22 hips, 1.1%) were missing. The baseline characteristics of the remaining 991 participants are shown in Table 1. Of the participants, 782 (78.9%) were female, the mean age was 55.9 years (SD 5.2), and mean BMI was 26.2 kg/m^2^ (SD 4.0). Of the 991 participants, 207 reported bilateral hip pain, 375 reported unilateral hip pain, and 409 reported no hip pain on either side. This resulted in 789 (39.8%) painful hips and 1193 (60.2%) pain-free hips.

**Table 1.** Baseline characteristics

**Characteristic**	**Participants (*N*= 991)^a^**
**Age, years, mean (SD)**	55.9 (5.2)

**Sex, female, *n* (%, 95% CI)**	782 (78.9, 76.4 to 81.4)

**BMI, kg/m^2^, mean (SD)**	26.2 (4.0)

**Ethnicity, White, *n* (%, 95% CI)**	965 (97.5, 96.5 to 98.5)

**Highest level of education, *n* (%, 95% CI)**	
Primary	186 (19.3, 16.8 to 21.8)
Secondary	437 (45.3, 42.2 to 48.4)
Higher	341 (35.4, 32.4 to 38.4)

**Hip pain, *n***	
No pain	409
Pain	582
Unilateral pain	375
Bilateral pain	207

**Morning stiffness in any hip, *n* (%, 95% CI)**	343 (35.9, 32.9 to 38.9)

**Knee pain in any knee, *n* (%, 95% CI)**	821 (82.8, 80.5 to 85.1)

**Duration of complaints, months, mean (SD)**	24.6 (24.2)

**Standardised WOMAC score, mean (SD)**	
Total (0–100)	24.6 (16.5)
Pain (0–100)	25.3 (17.2)
Stiffness (0–100)	33.1 (21.0)
Function (0–100)	23.5 (17.2)

**NRS hip and/or knee pain past week (0–10), mean (SD)**	3.6 (2.1)

**Using any pain medication, *n* (%, 95% CI)**	369 (38.0, 35.0 to 41.0)

a *Data missing for education (*n *= 27 individuals), ethnicity (*n *= 1), morning stiffness (*n *= 36), and pain medication (*n *= 19). BMI = body mass index. NRS = Numeric Rating Scale (in which 0 corresponds to no pain). SD = standard deviation. WOMAC = Western Ontario and McMaster Universities Osteoarthritis Index (in which 0 corresponds to no complaints).*

### Prevalence and association

The overall prevalence of definite hip ROA was 11.0% (95% CI = 9.6 to 12.4) (Table 2). When stratified by pain status, 13.3% (95% CI = 10.9 to 15.7) of all painful hips and 9.5% (95% CI = 7.8 to 11.2) of all pain-free hips showed definite hip ROA. The overall prevalence of early-stage hip ROA was 35.3% (95% CI = 33.2 to 37.4). Pain was prevalent in 41.2% (95% CI = 37.8 to 44.6) of painful hips and 31.4% (95% CI = 28.8 to 34.0) of pain-free hips.

**Table 2.** K&L grading among all hips and stratified by pain status^a^

**Characteristic**	**Total (*N*= 1982)**	**Painful hips (*n* = 789)**	**Pain-free hips ( *n*= 1193)**
**K&L grade, *n* (%)**			
0	1282 (64.7)	464 (58.8)	818 (68.6)
1	482 (24.3)	220 (27.9)	262 (22.0)
2	205 (10.3)	95 (12.0)	110 (9.2)
3	13 (0.7)	10 (1.3)	3 (0.3)
4	0 (0.0)	0 (0.0)	0 (0.0)

**Early-stage ROA, *n* (%, 95% CI)**	700 (35.3, 33.2 to 37.4)	325 (41.2, 37.8 to 44.6)	375 (31.4, 28.8 to 34.0)

**Definite ROA, *n* (%, 95% CI)**	218 (11.0, 9.6 to 12.4)	105 (13.3, 10.9 to 15.7)	113 (9.5, 7.8 to 11.2)

a *Early-stage ROA is defined as K&L grade ≥1 and definite ROA is defined as K&L grade ≥2. K&L = Kellgren and Lawrence. ROA = radiographic osteoarthritis.*

The odds ratio (OR) of painful hips having definite hip ROA was 1.51 in comparison with those with pain-free hips (95% CI = 1.16 to 1.98) (Table 3). The OR of people with painful hips having early-stage hip ROA was 1.47 (95% CI = 1.24 and 1.75) compared with those with pain-free hips (Table 4).

**Table 3.** Association between hip pain and definite hip ROA within sensitivity analyses

**Analyses**	**Painful hips included**	**Prevalence K&L ≥2 in painful hips, *n*/*N* (%)**	**Pain-free hips included**	**Prevalence K&L ≥2 in pain-free hips, *n*/*N* (%)**	**OR (95% CI)**
**Primary analysis**	All painful hips	105/789 (13.3)	All pain-free hips	113/1193 (9.5)	1.51 (1.16 to 1.98)
**Sensitivity analysis II**	All painful hips	105/789 (13.3)	Pain-free hips of participants without any hip pain	70/818 (8.6)	1.71 (1.19 to 2.46)
**Sensitivity analysis III**	Painful hips without concurrent knee pain	40/217 (18.4)	Pain-free hips without concurrent knee pain	11/117 (9.4)	2.30 (1.26 to 4.19)
**Sensitivity analysis IV**	Painful hips with concurrent knee pain	65/572 (11.4)	Pain-free hips with concurrent knee pain	102/1076 (9.5)	1.25 (0.91 to 1.72)

*K&L = Kellgren and Lawrence. OR = odds ratio. ROA = radiographic osteoarthritis.*

**Table 4.** Association between hip pain and early-stage hip ROA within sensitivity analyses

**Analyses**	**Painful hips included**	**Prevalence K&L ≥1 in painful hips, *n*/*N* (%)**	**Pain-free hips included**	**Prevalence K&L ≥1 in pain-free hips, *n*/*N* (%)**	**OR (95% CI)**
**Primary analysis**	All painful hips	325/789 (41.2)	All pain-free hips	375/1193 (31.4)	1.47 (1.24 to 1.75)
**Sensitivity analysis II**	All painful hips	325/789 (41.2)	Pain-free hips of participants without any hip pain	239/818 (29.2)	1.75 (1.37 to 2.23)
**Sensitivity analysis III**	Painful hips without concurrent knee pain	114/217 (52.5)	Pain-free hips without concurrent knee pain	52/117 (44.4)	1.49 (1.05 to 2.12)
**Sensitivity analysis IV**	Painful hips with concurrent knee pain	211/572 (36.9)	Pain-free hips with concurrent knee pain	323/1076 (30.0)	1.36 (1.12 to 1.66)

*K&L = Kellgren and Lawrence. OR = odds ratio. ROA = radiographic osteoarthritis.*

### Sensitivity analyses

When stratified by sex, the OR of hip pain for definite hip ROA was 1.74 (95% CI = 1.06 to 2.87) and 1.48 (95% CI = 1.06 to 2.06) for males and females, respectively. For early-stage hip ROA these ORs were 1.78 (95% CI = 1.23 to 2.59) and 1.44 (95% CI = 1.18 to 1.75), respectively. Results did not differ significantly between the sexes (see Supplementary Tables S1–S3). When participants with unilateral hip complaints were excluded, results did not differ. Finally, when including only participants with concurrent knee pain, the ORs of hip pain in those with definite and early hip ROA decreased to 1.25 (95% CI = 0.91 to 1.72) and 1.36 (95% CI = 1.12 to 1.66), respectively (Tables 3 and 4).

## DISCUSSION

### Summary

In this cross-sectional study, presence of hip pain was associated with both definite and early-stage radiographic hip OA in individuals who had recently or not yet presented to primary care. Nevertheless, the difference in prevalence of definite (13.3% versus 9.5%) and early-stage (41.2% versus 31.4%) hip ROA between painful and pain-free hips was small.

### Strengths and limitations

First, all participants were included based on the presence of knee or hip pain in at least one joint, so there was not a fully pain-free control group. In sensitivity analysis II, associations were examined with a control group consisting of only participants without any hip pain and with knee pain only. This showed only a marginal effect on the ORs, suggesting that hip pain is not associated with a higher likelihood of having hip ROA in the contralateral pain-free hip. However, in a prior study, knee pain was associated with hip ROA,^17^ which could indicate that the observed association in the current study might underestimate the true relationship in the population or that the OR of knee pain is simply different from contralateral hip pain.

Second, radiographic K&L scores were used in the current analyses that were read and scored with images paired and in known sequence.^26^ This approach is believed to produce more reliable and valid scores than those assigned to a single image with no follow-up images. Using this scoring approach, more hips were assigned K&L ≥2 compared with a single radiograph scoring approach.^26^ In primary care, the single scoring approach might resemble common practice most, as no follow-up radiographs are yet available. As hip ROA might be diagnosed less often using this approach, this suggests that the current results may overestimate the associations found in primary care. That said, the purpose of the current study was to understand the association between pain and ROA, and not to predict the prevalence of ROA in a clinical setting in which some cases of ROA might be missed.

Third, it should be noted that pain prevalence as an exposure for hip ROA was studied. Previous research suggests associations between pain severity and K&L ≥2,^30^ and between pain duration and joint space narrowing^31^ as well. Additionally, this study only evaluated K&L grades as outcomes. However, this might differ from the associations between hip pain and individual features of ROA (for example, joint space narrowing). Another limitation is that cross-sectional analyses were performed in this study, which prevented the evaluation of causal associations between pain and ROA. Future studies could evaluate whether the presence of pain predicts future onset or progression of structural OA features.

Finally, the participants were mainly of White ethnicity, therefore the external generalisability of the results might be limited. However, as mentioned, two of the few prior studies are Asian population-based studies and research studying the association in Europe and those of White ethnicity are scarce.^16^^,^^17^ As ROA prevalence differed within the Asian and European samples,^4^^,^^32^^–^^35^ the association between hip pain and radiographic hip OA within the White ethnic population might differ as well, and knowledge about this association is important.

### Comparison with existing literature

The observed association between pain and definite hip ROA is in contrast with several previous studies.^16^^,^^17^^,^^19^ Although two Asian population-based studies^16^^,^^17^ observed a relationship between hip pain and more severe hip ROA (K&L ≥3), no association was found between hip pain and definite hip ROA (K&L 2). This might be because of the different definite ROA definitions. Nevertheless, in these studies the painful ROA prevalence was low (*n* = 29, 0.75%^16^ and *n* = 26, 0.02%^17^), leading to imprecise results. Moreover, because of cultural and ethnic differences, and as the (symptomatic) hip ROA prevalence in Asia is lower than in Europe and the US, these associations might not be generalisable to the European population.^4^^,^^32^^–^^35^ One European study assessed the association between hip pain and moderate hip ROA (K&L 2–3).^19^ Although K&L grades 2 and 3 were combined, hip pain was not associated with hip ROA. Few symptomatic participants (*n* = 56) and only males were included in that analysis.

In the study by Jacobsen *et al*, hip pain was associated with definite hip ROA;^18^ however, data about uni- or bilateral hip pain were missing. Data on the side of the hip pain were not recorded and therefore the radiographically most affected hip was deemed symptomatic. This might have led to an over-estimation of the association between the prevalence of hip pain and ROA.

Previous research suggested that the association between hip pain and hip ROA has differed between the sexes.^16^^,^^17^ The current study did not find such a difference for definite and early-stage ROA; however, previous research showed that the association differed most in individuals with K&L ≥3, which was not analysed in the current study because of the low prevalence of more severe ROA in the participants (0.7%). Also, the current sample predominantly included females, which could cause the sex-stratified analyses to be underpowered.

The OA illness (symptoms) and disease (structural/radiographic changes) could be seen as two separate entities, as hip pain is not experienced in all patients with hip ROA and is present in many people without hip ROA.^19^^,^^35^ As a result of this structure–symptom discordance, reliable clinical diagnostic criteria to adequately initiate treatment when needed are important, but have not been established. Recent diagnostic criteria for early-stage hip OA have shown ‘poor’ to ‘fair’ diagnostic accuracy.^36^ The American College of Rheumatology (ACR) classification criteria were constructed for epidemiological purposes in secondary care and have shown poor reliability in primary care.^12^^,^^37^ The UK National Institute for Health and Care Excellence (NICE) diagnostic criteria are more applicable to primary care, but are primarily based on knee OA studies and have not been validated.^13^^,^^38^ When applied to the CHECK cohort, 62.7% of participants with hip complaints would be hip OA diagnosed according to the ACR criteria,^39^ whereas according to the NICE criteria all painful hips would be clinically diagnosed with OA — this, when only 13% of painful hips showed definite hip ROA in the current study. Because of their diagnostic purpose, the NICE criteria have a high sensitivity, but specificity is low. As a result, hips are diagnosed with OA, but the pain might be associated with another disease, such as low back pain or knee pain^17^ based on referred pain or through, for example, central sensitisation.^40^ Hip OA treatments might not be as effective for these hips.

The sensitivity analyses in the current study showed that in hips with concurrent knee pain the ORs for ROA decreased for definite ROA to an insignificant level, whereas the ORs in hips without concurrent knee pain increased. It is known that knee OA is associated with central sensitisation, which could lead to decreased pain thresholds.^40^^,^^41^ The difference between participants with and without knee pain might indicate the role of central sensitisation in patients presenting with multiple painful joints or might have been found as participants with knee pain or OA have an increased risk of having hip OA.^17^^,^^42^

### Implications for research and practice

As most people with painful hips did not have hip ROA and the association between hip pain and ROA was only moderate, radiographs likely do not help in the identification of patients with hip OA in primary care. This is in line with recent diagnostic criteria for early-stage hip OA in primary care, where the addition of radiographic variables did not increase diagnostic certainty.^36^ This suggests that prior guideline recommendations, mostly based on knee OA data, which state that radiographic evidence was not required for an OA diagnosis, apply for hip OA as well.^13^^,^^14^

An important question in these patients is whether the absence of radiographic evidence changes the treatment approach for patients who fulfil the clinical criteria for hip OA. Based on the results, the authors of the current study argue that a GP could — without radiographic confirmation of OA — consider these patients as having early OA and initiate appropriate treatments, such as education, exercise, and weight loss. Therefore, referral for radiography will not likely change clinical decision making in these patients.

In the CHECK cohort, no hip joint specific pain scores were collected at baseline. Hence, the influence of pain intensity in this association remains unknown. In knee OA pain, intensity was associated with knee ROA.^43^ Also in knee OA research, the association between pain and ROA strongly differed between cohort-level analyses and within-person analyses.^44^^,^^45^ In within-person analyses, knee pain was strongly associated with knee ROA. In hip OA, no research has been conducted to examine the association in a within-person design. Finally, although hip pain is of limited diagnostic value, it might still be of prognostic value for hip ROA. It is unknown whether hip pain is associated with an increased risk of hip ROA development. These uncertainties could be assessed in further research.

In conclusion, in patients recently or not yet presenting to primary care, hip pain was moderately associated with hip ROA and the difference in hip ROA prevalence between painful and pain-free hips was only modest. Therefore, radiographs provide GPs with little assistance to aid GPs in the identification of patients with hip OA. In hips that fulfil the clinical criteria of hip OA, GPs should consider starting the patient on guideline-recommended conservative OA treatments, without radiographic confirmation.

## Data

CHECK cohort data is freely available through the Dutch national centre of expertise and repository for research data at: www.dans.knaw.nl

## References

[1] Lespasio MJ, Sultan AA, Piuzzi NS (2018). Hip osteoarthritis: a primer. Perm J.

[2] Cross M, Smith E, Hoy D (2014). The global burden of hip and knee osteoarthritis: estimates from the global burden of disease 2010 study. Ann Rheum Dis.

[3] Pereira D, Peleteiro B, Araújo J (2011). The effect of osteoarthritis definition on prevalence and incidence estimates: a systematic review. Osteoarthritis Cartilage.

[4] Hunter DJ, Bierma-Zeinstra S (2019). Osteoarthritis. Lancet.

[5] Hunter DJ, Schofield D, Callander E (2014). The individual and socioeconomic impact of osteoarthritis. Nat Rev Rheumatol.

[6] Jordan KP, Jöud A, Bergknut C (2014). International comparisons of the consultation prevalence of musculoskeletal conditions using population-based healthcare data from England and Sweden. Ann Rheum Dis.

[7] van der Waal JM, Bot SD, Terwee CB (2006). The incidences of and consultation rate for lower extremity complaints in general practice. Ann Rheum Dis.

[8] Busija L, Bridgett L, Williams SR (2010). Osteoarthritis. Best Pract Res Clin Rheumatol.

[9] Dominick KL, Ahern FM, Gold CH, Heller DA (2004). Health-related quality of life and health service use among older adults with osteoarthritis. Arthritis Rheum.

[10] Gignac MA, Cott C, Badley EM (2000). Adaptation to chronic illness and disability and its relationship to perceptions of independence and dependence. J Gerontol B Psychol Sci Soc Sci.

[11] Hutchings A, Calloway M, Choy E (2007). The Longitudinal Examination of Arthritis Pain (LEAP) study: relationships between weekly fluctuations in patient-rated joint pain and other health outcomes. J Rheumatol.

[12] Altman R, Alarcón G, Appelrouth D (1991). The American College of Rheumatology criteria for the classification and reporting of osteoarthritis of the hip. Arthritis Rheum.

[13] National Institute for Health and Care Excellence (NICE) (2020). Osteoarthritis: care and management, CG177.

[14] Sakellariou G, Conaghan PG, Zhang W (2017). EULAR recommendations for the use of imaging in the clinical management of peripheral joint osteoarthritis. Ann Rheum Dis.

[15] Hall M, van der Esch M, Hinman RS (2022). How does hip osteoarthritis differ from knee osteoarthritis?. Osteoarthritis Cartilage.

[16] Iidaka T, Muraki S, Akune T (2016). Prevalence of radiographic hip osteoarthritis and its association with hip pain in Japanese men and women: the ROAD study. Osteoarthritis Cartilage.

[17] Park JH, Lee JS, Lee SJ, Kim YH (2021). Low prevalence of radiographic hip osteoarthritis and its discordance with hip pain: a nationwide study in Korea. Geriatr Gerontol Int.

[18] Jacobsen S, Sonne-Holm S, Søballe K (2004). Radiographic case definitions and prevalence of osteoarthrosis of the hip: a survey of 4151 subjects in the Osteoarthritis Substudy of the Copenhagen City Heart Study. Acta Orthop Scand.

[19] Birrell F, Lunt M, Macfarlane G, Silman A (2005). Association between pain in the hip region and radiographic changes of osteoarthritis: results from a population-based study. Rheumatology (Oxford).

[20] James KA, von Heideken J, Iversen MD (2021). Reporting of adverse events in randomized controlled trials of therapeutic exercise for hip osteoarthritis: a systematic review. Phys Ther.

[21] Gazendam A, Ekhtiari S, Bozzo A (2021). Intra-articular saline injection is as effective as corticosteroids, platelet-rich plasma and hyaluronic acid for hip osteoarthritis pain: a systematic review and network meta-analysis of randomised controlled trials. Br J Sports Med.

[22] Yang W, Sun C, He SQ (2021). The efficacy and safety of disease-modifying osteoarthritis drugs for knee and hip osteoarthritis — a systematic review and network meta-analysis. J Gen Intern Med.

[23] Wesseling J, Boers M, Viergever MA (2016). Cohort profile: Cohort Hip and Cohort Knee (CHECK) study. Int J Epidemiol.

[24] Wesseling J, Dekker J, van den Berg WB (2009). CHECK (Cohort Hip and Cohort Knee): similarities and differences with the Osteoarthritis Initiative. Ann Rheum Dis.

[25] Kellgren JH, Lawrence JS (1957). Radiological assessment of osteo-arthrosis. Ann Rheum Dis.

[26] Macri EM, Runhaar J, Damen J (2021). Kellgren & Lawrence grading in cohort studies: methodological update and implications illustrated using data from the CHECK cohort. Arthritis Care Res (Hoboken).

[27] Epitools. Sample size calculations.

[28] Schiphof D, Kerkhof HJ, Damen J (2013). Factors for pain in patients with different grades of knee osteoarthritis. Arthritis Care Res (Hoboken).

[29] Szilagyi IA, Waarsing JH, Schiphof D (2022). Towards sex-specific osteoarthritis risk models: evaluation of risk factors for knee osteoarthritis in males and females. Rheumatology (Oxford).

[30] Pereira D, Severo M, Santos RA (2016). Knee and hip radiographic osteoarthritis features: differences on pain, function and quality of life. Clin Rheumatol.

[31] Bierma-Zeinstra SM, Oster JD, Bernsen RM (2002). Joint space narrowing and relationship with symptoms and signs in adults consulting for hip pain in primary care. J Rheumatol.

[32] Hoaglund FT, Steinbach LS (2001). Primary osteoarthritis of the hip: etiology and epidemiology. J Am Acad Orthop Surg.

[33] Fransen M, Bridgett L, March L (2011). The epidemiology of osteoarthritis in Asia. Int J Rheum Dis.

[34] Kim C, Linsenmeyer KD, Vlad SC (2014). Prevalence of radiographic and symptomatic hip osteoarthritis in an urban United States community: the Framingham osteoarthritis study. Arthritis Rheumatol.

[35] Kim C, Nevitt MC, Niu J (2015). Association of hip pain with radiographic evidence of hip osteoarthritis: diagnostic test study. BMJ.

[36] Runhaar J, Özbulut Ö, Kloppenburg M (2021). Diagnostic criteria for early hip osteoarthritis; first steps, based on the CHECK study. Rheumatology (Oxford).

[37] Reijman M, Hazes JM, Koes BW (2004). Validity, reliability, and applicability of seven definitions of hip osteoarthritis used in epidemiological studies: a systematic appraisal. Ann Rheum Dis.

[38] Kinds MB, Welsing PM, Vignon EP (2011). A systematic review of the association between radiographic and clinical osteoarthritis of hip and knee. Osteoarthritis Cartilage.

[39] Damen J, van Rijn RM, Emans PJ (2019). Prevalence and development of hip and knee osteoarthritis according to American College of Rheumatology criteria in the CHECK cohort. Arthritis Res Ther.

[40] Woolf CJ (2011). Central sensitization: implications for the diagnosis and treatment of pain. Pain.

[41] Fingleton C, Smart K, Moloney N (2015). Pain sensitization in people with knee osteoarthritis: a systematic review and meta-analysis. Osteoarthritis Cartilage.

[42] Prieto-Alhambra D, Judge A, Javaid MK (2014). Incidence and risk factors for clinically diagnosed knee, hip and hand osteoarthritis: influences of age, gender and osteoarthritis affecting other joints. Ann Rheum Dis.

[43] Duncan R, Peat G, Thomas E (2007). Symptoms and radiographic osteoarthritis: not as discordant as they are made out to be?. Ann Rheum Dis.

[44] Neogi T, Felson D, Niu J (2009). Association between radiographic features of knee osteoarthritis and pain: results from two cohort studies. BMJ.

[45] Wang K, Kim HA, Felson DT (2018). Radiographic knee osteoarthritis and knee pain: cross-sectional study from five different racial/ethnic populations. Sci Rep.

